# Genetic Polymorphisms of Xenobiotic Metabolizing Genes (*GSTM1*, *GSTT1*, *GSTP1*), Gene-Gene Interaction with Association to Lung Cancer Risk in North India; A Case Control Study

**DOI:** 10.31557/APJCP.2019.20.9.2707

**Published:** 2019

**Authors:** Ritam bhara, Sonia Tiwari, Sivakumar Vijayaraghavalu, Munish Kumar

**Affiliations:** 1 *Department of Biochemistry, University of Allahabad, *; 2 *Department of Radiation Oncology, Kamala Nehru Memorial Hospital, Allahabad, UP-211002,*; 3 *Central Research Facility, Sri Ramachandra Institute of Higher Education and Research (Deemed to be University), Porur, Chennai, 600116, Tamil Nadu, India. *

**Keywords:** GSTs, Polymorphism, GSTT1, GSTM1, GSTP1, Lung cancer

## Abstract

**Aim:**

In this case control study involving, 220 human subjects; polymorphisms in xenobiotic metabolizing genes (*GST-M1*, *-T1* and -*P1*) and their association to lung cancer risk is being analysed among smokers and non-smokers. *GSTM1* or *GSTT1* gene polymorphism and amino acid changes in *GSTP1* have been correlated and may be associated to lung cancer risk. Other factor includes exposure to environmental pollutants and life style choices. We have explored gene-gene and gene-environment interaction in the aetiology of lung cancer risk among north Indian population.

**Patients and Methods:**

For the study we have collected 120 lung cancer patient blood samples from Kamala Nehru Memorial Cancer Hospital, Allahabad, Uttar Pradesh and 100 matched controls. DNA was isolated and *GST-M1* and - *T1 *genotyping were assessed by multiplex PCR whereas the *GSTP1* polymorphism was analysed using restriction fragment length polymorphism. The risk of lung carcinogenesis was assessed using logistic regression analysis calculating the odd ratio (OR) with 95% confidence interval (CI).

**Results:**

The risk of lung carcinogenesis was three fold higher for null *GSTT1* (OR=3.045, 95%CI=1.750-5.301, p-value <0.001) genotype; whereas other two types; *GSTM1* (OR= 1.342, 95% CI=0.788-2.284, p-value=0.270) and *GSTP1* (OR=0.806, 95% CI=0.526-1.236, p-value=0.323) showed no association to lung cancer susceptibility respectively. Smokers diagnosed with lung cancer had more null genotypes for GSTT1 (OR=4.773, 95%CI=1.939-11.751, p<0.001). The ‘at risk’ genotype combination GSTM1 (null) /*GSTT1* (null) (OR=1.76, 95%CI; 0.920-3.370, p-value=0.03) showed increased susceptibility to lung cancer risk. The genotype combination of *GSTT1* (null)/*GSTP1* (Ile/Ile) (p=0.009) was associated with increased lung cancer risk.

**Conclusion:**

The results of this study suggest that; GSTT1 null genotype were more susceptible for lung cancer risk and smoking increases the susceptibility for lung cancer several folds among the North Indian population. Gene-gene interaction for null genotypes of *GSTM1* and *GSTT1* were correlated with higher risk of having lung cancer.

## Introduction

Lung cancer is widespread malignancy in both men/women and reported increasing incidence rate and mortality among all cancer types across the world including India in last few decades. It is ranked fourth for new cases incidence and ranked third in number of death due to cancers (GLOBOCAN, WHO, 2018). Incidence of lung cancer in both sexes in Asia is 58.5%, mortality 60.7% and 5 year prevalence is 56.6% which is higher than other cancers worldwide. New cases of lung cancer in worldwide outlines in 2018 in both sexes and all ages are 11.6% (2,093,876) in total reported 18,078,957 cases (GLOBOCAN, WHO, 2018). Death cases reported according to GLOBOCAN 2018 data for both sexes and all ages are up to 18.4% (1,761,007) which is significantly higher in terms of mortality rate among all cancers. Lung cancer basically comprises of two types; non-small cell lung cancer (NSCLC) and small cell lung cancer (SCLC) in which 80% of occurrence is reported for NSCLC. Tobacco smoking is one of the leading causes for lung cancer related risks, but only 5-10% smokers develop lung cancer in their life time. The various carcinogens present in tobacco and cigarette smoke are polycyclic aromatic hydrocarbons, benzo[α] pyrene, ethylene oxide, aldehydes and nitrosamine. These aromatic carcinogens are detoxified by cytochrome P450 gene and GSTs gene super family (Clement-Duchene et al., 2010, Garte et al., 2007). Detoxification of carcinogens occurs in sequential phases includes phase I and phase II detoxification enzymes. Phase I detoxification enzymes makes catalytically active pro-carcinogens mediated through cytochrome P450 super gene family (Kiyohara et al., 2012). The GSTs (glutathione S-transferase) - *GSTM1*, *GSTT1*, and *GSTP1* plays prominent role in metabolic detoxification, they are classified as phase II detoxification enzymes (Malik et al., 2010). They execute their role through conjugation to electrophilic metabolite with reduced glutathione. The carcinogens when not biotransformed gains ability to become activated consequently, forms DNA adduct which hampers genomic integrity leading to carcinogenesis. *GSTM1* and *GSTT1* are both expressed in lung tissues and play a crucial role in detoxification of cigarette smoke in lungs. The impaired functioning of null genotypes of *GSTM1* and *GSTT1* is unable to prevent formation of DNA adducts and subsequently leading to promotion of carcinogenesis. DNA adducts level found to be significantly increased with association to gene polymorphism of *GSTT1*, *GSTM1 *and *GSTP1 *(Lee et al., 2010). *GSTP1* has nucleotide transition from A to G in exon 5 (codon 105) occur changes in isoleucine (Ile) to valine (Val) affects the enzymatic activity and increased susceptibility towards cancer (Mota et al., 2015, Vettriselvi et al., 2006). *GSTP1* functions in detoxification of compounds known to cause oxidative stress like thymidine and uracil propanol (Berhane et al., 1994). Studies on GSTs gene polymorphisms among North Indian population are limited and inconsistent findings obtained across Indian region. So there is need to explore gene-gene and gene-environment interaction to find its association to lung cancer risk. We hypothesized in present case control study that deletion polymorphism of *GSTT1* and *GSTM1 *higher in lung cancer patients compared to controls as well as with relation to smoking. We have also explored *GSTP1 *genotypes distribution in cases and controls whether there is more susceptibility towards lung cancer risk of other alleles and genotypes.

## Materials and Methods 

*Study Subjects*

In this study both the cancer patients (n=120) and matched controls (without cancer; n=100) were recruited from Kamala Nehru Memorial Cancer Hospital, Uttar Pradesh, India. Based on the bronchoscopy and histological studies from the lung biopsy; the cancer patients were divided into following; adenocarcinoma (n=76), squamous cell carcinoma (n=28) and small cell lung carcinoma (n=16) according to medical records of patients. The ethnic North Indians who were unrelated to cancer patients served as controls. The study was conducted (February 2017 to February 2019) post approval from Population Resource and Research centre, Allahabad Institute Ethical Committee (IERB Reference: 18/9.39) and with prior consent from the participants. Also, ethical principles as stated in Declaration of Helsinki were strictly followed while handling human subjects. Questionnaires for the control and cancer patients were prepared both in local language and English. It includes ethnicity, smoking habits, dietary habits, socio-economic status, occupation and familial history. The lung cancer patients were recruited without any prior neoadjuvant therapy. The sample size taken for this study was appropriate to strengthen 80% statistical power for allele frequencies ranges between 11-35% to estimate odd ratios greater than 1.34 or lower than 0.69 for finding risk associated to lung cancer.

Inclusion and exclusion criteria for lung cancer patients and controls are as follows:

*Inclusion criteria*

- Ethnic North Indians in the age range of 18 to 75 with lung cancers. 

- Patients smoking ≥ 10 cigarettes/day for at least 20 years. 

- Non-smoking cancer patients those who never smoked less than a one pack year or 20 cigarettes per day.

- Patients undergone bronchoscopy.

- Age, sex and ethnicity matched healthy individuals without reported any kinds of cancer (s) were included as controls. 

*Exclusion criteria*

Men and women greater than 75 years and/or with double metastasis and/or diagnosed with any other diseases or disorders were excluded from the study. As well both the cancer patients and controls that were not willing to participate in the study were excluded.

**Figure 1:**
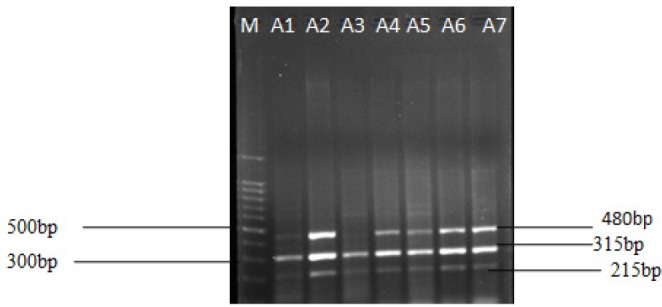
Multiplex PCR for *GSTT1* and *GSTM1*. 2% gel electrophoresis of *GSTM1* and *GSTT1 *genotype. Lane M, base pair marker (ProxiO, DNA marker). Lane A1 and A3 represents deletion of *GSTT1 *(480bp), Lane A1 to A7 showed presence of *GSTM1* (215bp), *CYP1A1* (315) serves as internal control

**Figure 2:**
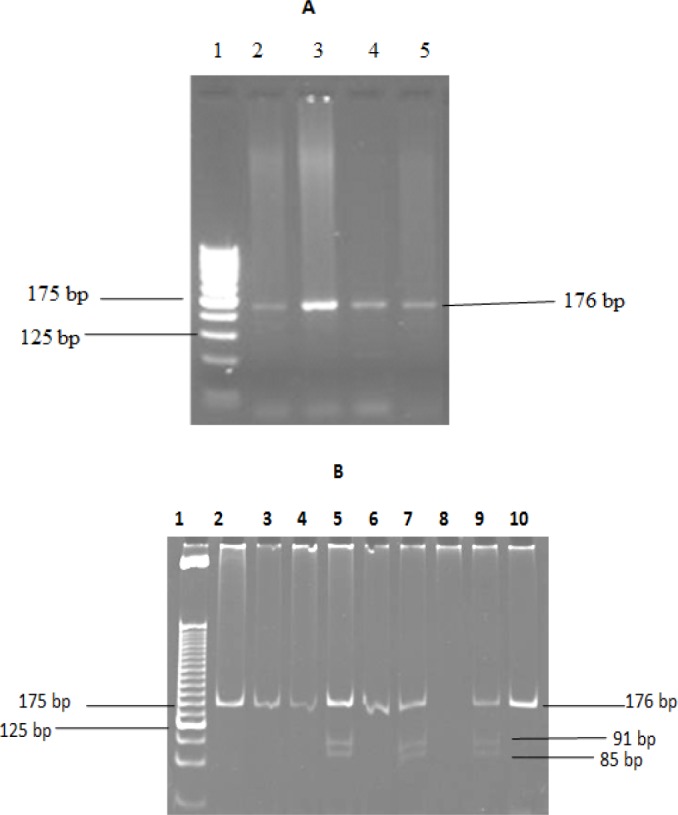
Genetic Polymorphism Using Restriction Fragment Length Polymorphism, Native 10% PAGE, RFLP Products of *GSTP1*. A, Lane 1 showed 25bp gene marker, lane 2, 3, 4 and 5 showed 176 bp PCR products (2% agarose gel electrophoresis); B, Lane 1 showed 25bp gene marker, Lane 2, 3, 4,6 and 10 (Ile/Ile) genotype and Lane 5, 7, 9 (Ile/Val) genotype RFLP product (Native 10% PAGE)

**Table 3:** Genotype and Allele Frequency with Odd Ratio of GSTP1

*GSTP1*	AA	AG	GG	P-value	OR (95%CI)
Genotype Frequency					
Controls n=100 (%)	52 (52.0)	41(41.0)	7 (7.0)	1.0	1.0
Cases = 120 (%)	72 (60)	40 (33.34)	8 (6.67)	0.323	0.806 (0.526 – 1.236)
Chi square (ᵡ^2^)= 1.499		P- value = 0.473			
Allele Frequency	A	G	p-value	OR (95%CI)	
Controls (%)	145 (72.5)	55 (27.5)	1.0	1.0	
Cases (%)	184 (76.67)	56 (23.34)	0.517	0.865 (0.559- 1.340)	

**Table 4:** Correlation of Smokers and Non-Smokers Status to *GSTM1*, *GSTT1* and *GSTP1 *Genotype, *All p-values <0.05 was Considered Statistically Significant

Gene	Smokers Cases (%)	Controls (%)	OR (95%CI)	P -value
*GSTM1*				
Present	30 (26.78)	11 (42.30)	1.0	1.0
Absent	82 (73.21)	15 (57.69)	2.00 (0.829-4.848)	0.123
*GSTT1*				
Present	22 (19.64)	14 (53.84)	1.0	1.0
Absent		12 (46.15)	4.773 (1.939-11.751)	<0.001
*GSTP1*	90 (80.35)			
AA	72 (64.28)	10 (38.46)	1.0	1.0
AG	37 (33.03)	15 (57.69)	0.434 (0.206-0.916)	0.029
GG	3 (2.67)	1 (3.84)		
	Non-Smokers			
*GSTM1*	Cases (%)	Controls (%)	OR (95% CI)	P-value
Present	4 (50)	54 (72.97)	1.0	1.0
Absent	4 (50)	20 (27.02)	2.7 (0.616-11.835)	0.188
*GSTT1*				
Present	6 (75)	59 (79.72)	1.0	1.0
Absent	2 (25)	15 (20.27)	1.311 (0.240-7.160)	0.754
*GSTP1*				
AA	4 (50)	44 (59.45)	1.0	1.0
AG	3 (37.5)	28 (37.83)	1.739 (0.523- 5.785)	0.367
GG	1 (12.5)	2 (2.70)		

**Table 5:** Genotype Combinations of *GSTM1*, *GSTT1 *and *GSTP1*, *All p-values <0.05 was Considered Statistically Significant

Genotype Combination		Control (n %)	Cases (n %)	OR (95% Confidence interval)	P –value
*GSTM1*	*GSTT1*				
Positive	Positive	54 (54)	48 (40)	1.0	1.0
Null	Null	46 (46)	72 (60)	1.761 (1.030-3.012)	0.039*
Positive	Null	54 (54)	72 (60)	1.5 (0.887-2.5360	0.130
Null	Positive	46 (46)	48 (40)	1.174 (0.670-2.057)	0.575
*GSTM1*	*GSTP1*				
Positive	Ile/Ile	54 (54)	72 (60)	1.0	1.0
Null	Ile/Val+Val/Val	46 (46)	48 (40)	0.783 (0.458-1.339)	0.371
Null	Ile/Ile	46 (46)	72 (60)	1.174 (0.704-1.957)	0.539
Positive	Ile/Val+ Val/Val	54 (54)	48 (40)	0.667 (0.394-1.127)	0.130
*GSTT1*	*GSTP1*				
Positive	Ile/Ile	67 (67)	72 (60)	1.0	1.0
Null	Ile/Val+Val/Val	33 (33)	48 (40)	1.354 (0.778-2.356	0.284
Null	Ile/Ile	33 (33)	72 (60)	2.030 (1.196-3.448)	0.009*
Positive	Ile/Val+ Val/Val	67 (67)	48 (40)	0.667 (0.405-1.097)	0.111
*GSTP1*	GSTM1				
Ile/Ile	Positive	52 (52)	48 (40)	1.0	1.0
Ile/Val+Val/Val	Null	48 (48)	64 (53.34)	1.444 (0.840-2.484)	0.184
Ile/Ile	Null	52 (52)	64 (53.34)	1.333 (0.780-2.280	0.293
Ile/Ile+ Val/Val	Positive	48 (48)	56 (46.67)	1.167 (0.677-2.011)	0.579
GSTP1	*GSTT1*				
Ile/Ile	Positive	52 (52)	48	1.0	1.0
Ile/Val+Val/Val	Null	48 (48)	72 (60)	1.625 (0.951-2.777)	0.076
Ile/Ile	Null	52 (52)	72 (60)	1.5 (0.883-2.549)	0.134
Ile/Ile+ Val/Val	Positive	48 (48)	48 (40)	1.083 (0.619-1.897)	0.779

*DNA extraction and genotyping*

2ml of peripheral blood samples withdrawn for genomic DNA isolation, using Qaigen (Germany) DNA blood mini isolation kit following the manufacturers’ protocol and quantified on nano-drop spectrophotometer (Agilent technologies, Santa Clara, USA) from both cancer patients and control subjects. The DNA samples were stored at -80 ^o^C until further use. 

*GSTM1 and GSTT1 genotyping*

Multiplex PCR was performed to analyse null genotypes of *GSTM1* and *GSTT1* gene polymorphism as described earlier (Rehman et al., 1996). Briefly, to each well in a PCR plate, 25 µl (2X PCR master mixes, Sigma Aldrich, St. Louis, USA), 50-100 ng of DNA and 10 pmol of primers were added and the reaction was set with its respective positive and negative controls in a thermal cycler 8800 (Agilent Technologies, Australia). PCR cycling conditions: initial denaturation at 94 ^o^C for 5 min, followed by denaturation 94 ^o^C for 1 min, annealing 59 ^o^C for 1 min and extension 72 ^o^C for 10 min. The PCR products were then electrophoresed using 2% agarose gel stained with ethidium bromide and visualized using Geldoc XR+ (Biorad System, Canada) system. Bands at 215- and 480 bp were considered as positive for *GSTM1* and *GSTT1 *genotypes respectively, while CYPA1 yielded band of 315 bp (Figure 1). 

*Primer sequences used were as follows*

**Figure:**
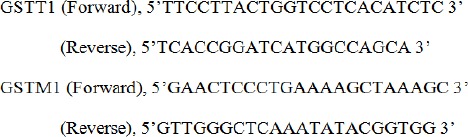


*GSTP1 genotyping*

GSTP1genotyping performed by PCR-RFLP method as described earlier (Harries et al., 1997) using following primer sequences: 

Forward, 5’-ACCCCAGGGCTCTATGGGAA-3’; 

Reverse, 5’-TGAGGGCACAAGAAGCCCCT-3’

The PCR conditions used were as follows: Initial denaturation at 95^o^C for 5 min, followed with denaturation of 94^o^C for 30 sec, annealing 58 ^o^C for 45 sec for 35 cycles and final extension at 72 ^o^C for 10 min. The amplified product (176bp) was digested by incubating overnight with BsmA1 (New England Biolabs, USA) at 37 ^o^C and electrophoresed using 10% polyacrylamide gel. The amplified PCR product of 176bp obtained for *GSTP1* by 2% gel electrophoresis (Figure 2A). After performing 10% native PAGE ; the band pattern showed 176bp undigested product obtained for Ile/Ile (wild type) genotype, 91 and 85 bp genotype showed mutant genotype Val/Val and three fragment obtained for Ile/Val genotype 176, 91 and 85 bp respectively(Figure 2 B). Then the ethidium bromide staining was done and visualized using Geldoc system (Bio Rad system, Canada).

*Statistical analysis*

The gene polymorphisms of *GSTT1*, *GSTM1*, *GSTP1 *distribution in North Indian population assessed by unconditional logistic regression used for evaluation of odd ratio (OR) and 95% confidence interval (CI) used to describe the strength of association with disease. Binary Logistic Regression model used to evaluate the difference in prevalence and association between cases and groups. p-value < 0.05 was considered as statistically significant. Statistical analysis was performed using SPSS software 16.00 (SPSS In; Chicago, IL, USA). 

## Results

In present study we assessed the role of xenobiotic metabolizing genes, age, gender, smoking and histology with relation to lung cancer risk. The age of lung cancer cases (mean±SD, 58.41±10.49, Table 1) including 104 males and 16 females found comparatively higher than controls (51.51±10.76) and statistically significant in both gender (Table 1). The histology data obtained from medical records of lung cancer patients having squamous cell carcinoma (SCC, 23.34%), adenocarcinoma (ADCC, 63.34%) and small cell carcinoma (SCLC, 13.34%) (Table 1). It was observed in present study that most of lung cancer patients have TNM staging I (56.67%) and II (28.34%) (Table 1). Out of 120 lung cancer patients 93.3% were smokers and 6.67% non-smokers which was significantly higher than that of controls (Table 1). The genotype frequencies in lung cancer patients obtained for *GSTT1* (positive, 40%), GSTT1 (null, 60%) and in controls GSTT1 (positive, 67%), GSTT1 (null, 33%) (Table 2). GSTT1 null (60%) genotype of lung cancer cases were higher comparatively to controls (33%, Table 2). We found increased *GSTM1* deletion polymorphism in lung cancer cases (53.34%) than controls (46%, Table 2). The odd ratios found for* GSTT1* (OR=3.04, CI=1.750-5.301, p<0.001, Table 2) showed strong association whereas *GSTM1 *(OR=1.342, CI=0.788-2.284, p=0.270, Table 2) showed no association. The null genotype for both *GSTM1* and *GSTT1* with estimated odd ratio (OR=1.76, 95% CI: 1.030-3.012, p=0.03, Table 5) found to be statistically significant in lung cancer patients. The odd ratio estimated for smokers in lung cancer cases with association to *GSTT1* null genotypes (OR=4.773, 95%CI=1.939-11.751, p<0.001) found statistically significant whereas no association found with *GSTM1* (OR=2.00, 95%CI=0.166-1.26, p-value=0.123, Table 4). In smokers having lung cancer both GSTT1 (80.35%) and *GSTM1* (73.21%) null genotypes were higher than controls of *GSTT1* (46.15%) and *GSTM1* (57.69%) (Table 4). The genotype frequency of GSTP1 (OR=0.806, 95%CI=0.526-1.236 p=0.323, Table 3) showed no association to lung cancer risk. In cases genotype frequency for* GSTP1*; AA (60%), AG (33.34 %), GG (6.67%) in which AG (Ile/Val, 41%) was lower in lung cancer cases comparatively to controls (Table 3). Allele frequencies of lung cases in GSTP1 showed that allele A (76.67%) higher than controls (72.5%) and allele G (23.34%) lower than controls (27.5%) (Table 3). The genotype AG (Ile/Val) of* GSTP1* found to be lower in smokers of lung cancer cases (33.03%, Table 4) than controls (57.69%, Table 4). *GSTP1* genotype showed no association to smoking (OR=0.434, 95% CI=0.206-0.916 p-value=0.02, Table 4). The genotype combination of* GSTT1* (null) and *GSTP1* (Ile/Ile) showed significant association (OR=2.030, 95%CI=1.196-3.448, p-value=0.009, Table 5). Genotype combinations of *GSTM1* (positive) /*GSTT1* (null) (OR=1.5, 95% CI=0.887-2.5360) and *GSTM1* (null)/*GSTT1* (positive) (OR=1.174, 95%CI=0.670-2.057) (Table 5) were associated with lung cancer risk but statistically non significant. 

Genotypic combination of *GSTP1*(Ile/Val+Val/Val) /*GSTM1*(null) (OR=1.45, 95%=0.840-2.484), *GSTP1*(Ile/Ile)/*GSTM1*(null)(OR=1.34,95%CI=0.780-2.280), GSTP1(Ile/Ile+Val/Val)/*GSTM1*(positive) (OR=1.167,95%CI=0.677-2.011), GSTP1(Ile/Ile)/GSTT1 (null) (OR=1.5, 95%CI=0.883-2.549) and *GSTP1* (Ile/Val+Val/Val)/*GSTT1*(null) (OR=1.62,95% CI=0.951-2.76) (Table 5) showed association but statistically non significant. *GSTM1* (null)/*GSTP1* (Ile/Ile) (OR=1.174, 95%CI=0.704-1.957), *GSTT1* (null)/*GSTP1 *(Ile/Val+Val/Val) (OR=1.354, 95%CI=0.778-2.356) (Table 5) correlated to lung cancer risk and statistically non- significant. 

## Discussion

GSTs are phase II metabolizing enzymes, it consist of several forms of GSTs such as GSTA (alpha), *GSTT1 *(theta), *GSTM1* (mu) and GSTP1 (pi), and have role in biotransformation of carcinogens. They are the candidate genes to detoxify carcinogenic compounds and thus show susceptibility towards lung cancer risk because of their impaired ability (Ihsan et al., 2014). The function of all GSTs is to metabolize the carcinogens via conjugating to glutathione and then converting into hydrophilic metabolites and eliminating it from the body. The genotype frequency of these GSTs varies in Indian subcontinents. In present study, we have assessed the role of GSTs polymorphism with association to lung cancer risk with three polymorphic genotypes of *GSTT1*, *GSTM1*, and *GSTP1*. Our findings suggested that the GSTT1 holds positive association towards lung cancer risk whereas *GSTM1* and *GSTP1* showed no association to lung cancer risk in this study. The genotype combination of *GSTM1* (null)/*GSTT1* (null) and *GSTT1* (null)/*GSTP1* (Ile/Ile) showed increased susceptibility towards lung cancer. In present study the age mean differences between lung cancer cases and control were higher suggesting that it is an old stage disease in which DNA adducts accumulation occurs due to smoking and environmental pollutants exposure develops lung cancer risk as impaired functions of GSTs. In normal individuals of south Indian population frequency of deletion for *GSTT1* is reported as (30.4%) and *GSTM1* (16.8%) whereas in present north Indian population study null genotypes of *GSTT1* and *GSTM1* reported in control subjects 33% and 46% respectively (Naveen et al., 2004). Bag et al., (2014) showed in North Indian population increased susceptibility of lung cancer patients having *GSTT1* null genotypes in cases (23%) than controls (15%), similarly we also found increased frequency of *GSTT1* null genotypes 60% in cases than (33%) controls, and these results suggest that ethnicity and life style choice have prominent role in aetiology of lung cancer. We also found no association (p-value=0.367) with *GSTP1 *Ile/Val genotype in non- smokers similar to North Eastern region of India where it was found same with non-smokers (Ihsan et al., 2011).

Recent study in Iranian population showed strong correlation of GSTT1 (OR=2.4, 95%CI=1.32-4.35, p-value=0.005) and no association to* GSTM1* (OR=1.33, 95%CI=0.79-2.25, p-value=0.35) similar to our findings in north Indian population (Adibhesami et al., 2018). Our study showed positive association to lung cancer risk with genotype combination of *GSTM1* (null) and *GSTT1* (null) (OR=1.76; 95% CI=1.030-3.012), showed previously by Cajas-Salazar et al., 2003 (OR=2.32; CI=1.01-6.04). In Caucasian population Lewis et al., 2002 showed lower lung cancer risk with association to *GSTM1* null genotype (OR=0.50; 95% CI=0.29-0.87) similar to our results showed decreased association in North Indian population (OR=1.34; 95% CI=0.788-2.284). Researchers from North Eastern region of India found no association to* GSTT1* (OR=0.32, 95%CI=0.15-0.71, p-value=0.13) contrary to our result we found strong association to *GSTT1 *(OR=3.045, 95%CI= 1.750-5.301, p-value <0.001). Both the regions of India have different geography, climate and life style differences pose strong effect of GSTs gene polymorphism in lung cancer risk (Ihsan et al., 2014).

Shukla et al., (2013) reported significant association in north Indian population *GSTT1* null genotypes (OR=1.87, 95% CI: 1.25-2.80, p=0.002) whereas GSTM1 null genotypes (OR=1.03, CI: 0.71-1.51, p=0.875) which was consistent to present study. 

Our study finds strong correlation with genotype combination of *GSTM1* (null)/*GSTT1* (null) (p-value=0.03) and* GSTT1* (null)/*GSTP1* (Ile/Ile) (p-value=0.009) showed similarity to Chen et al., 2006 reported values *GSTM1* (null)/*GSTT1* (null) (p-value=0.000), *GSTP1* (Ile/Val+Val/Val) and* GSTT1* (null) (p-value=0.003). 

A study in north Indian population by Sharma et al., (2015) found that ‘at risk’ genotypes of null* GSTM1* and *GSTT1* are strongly associated to lung cancer risk which was in favour of our findings. The genotype combination of null *GSTT1* and *GSTP1* Ile/Ile genotypes (p=0.0001) have two times increased risk of having lung cancer similar to our study. They found* GSTM1* null genotypes (OR=1.65, 95% CI=116-2.3) was associated to lung cancer risk whereas we found no association. Similar to our study Jiang et al., (2014) showed null genotype of *GSTT1* (OR=1.574, 95%CI=1.044-2.372, p-value=0.03) in smokers are positively correlated to lung cancer risk and in our study *GSTT1* (OR=4.773 95%CI=1.930-11.751, p<0.001) associated with four fold increased risk of having lung cancer in North Indian population. 

In contradictory to our study Nasir Uddin et al., (2014) reported no association of *GSTT1* (OR=0.84, 95% CI: 0.46-1.55, p=0.573) and *GSTM1* (OR=1.06, 95% CI: 0.62-1.81, p=0.820) as we find association with *GSTT1 *genotypes whereas they found strong association of *GSTP1* (OR=3.56, 95%CI: 1.70-7.46, p=0.0001) and we found no association. In similarity to our results, South Indian study confirmed two fold increased risk of having lung cancer with *GSTT1* null genotypes (Peddireddy et al., 2016). The limitation of the present study is that it’s small sample size and few genes chosen for study. 

The main outcome of this study suggested that *GSTT1 *was the prominent candidate gene and has three-fold increased susceptibility to lung cancer risk in North Indian population. Different genotype combinations posing positive correlation to promote lung cancer risk. Genotype combination of *GSTM1* (null)/*GSTT1* (null) and *GSTT1* (null)/*GSTP1* (Ile/Ile) considered to be ‘at risk’ genotypes showed strong association towards risk of lung cancer in north Indian population. They can be taken into account for detection for lung cancer tumorigenesis and overall risk factor generation. It can serve as prognostic marker in lung carcinogenesis. There are various conflicting results obtained in various population of Indian population which showed that life style choices and environmental exposure are key players in posing lung cancer risk. 
